# The significance of ENAH in carcinogenesis and prognosis in gastric cancer

**DOI:** 10.18632/oncotarget.19801

**Published:** 2017-08-02

**Authors:** Dan-Dan Wang, Qun Jin, Lei-Lei Wang, Shu-Fang Han, Yi-Bing Chen, Guo-Dong Sun, Shi-Fei Sun, Shu-Wang Sun, Tao Wang, Fan-Jie Liu, Ping Wang, Bin Shi

**Affiliations:** ^1^ Shandong Medicinal Biotechnology Centre, Key Laboratory for Rare & Uncommon Diseases of Shandong Province, Back and Neck Pain Hospital of Shandong Academy of Medical Sciences, Jinan 250062, People's Republic of China; ^2^ The General Hospital of Jinan Military Command, Jinan 250012, People's Republic of China; ^3^ Key Laboratory for Applied Microbiology of Shandong Province, Ecology Institute of Shandong Academy of Sciences, Jinan 250014, People's Republic of China; ^4^ Center of Genetic & Prenatal Diagnosis, First Affiliated Hospital, Zhengzhou University, Zhengzhou 450052, People's Republic of China; ^5^ Affiliated Hospital of Shandong Academy of Medical Sciences, Shandong Academy of Medical Sciences, Jinan 250031, People's Republic of China; ^6^ School of Precision Instrument and Opto Electronics Engineering, Tianjin University, Tianjin 300072, People's Republic of China; ^7^ Shandong Academy of Chinese Medicine, Jinan 250014, People's Republic of China

**Keywords:** gastric adenocarcinoma, ENAH, expression, prognosis, function

## Abstract

The ENAH gene, which encodes a member of the enabled/vasodilator-stimulated phosphoprotein (Ena/VASP) family of proteins, is involved in the assembly of actin filaments required for cell adhesion and motility. Recent studies show overexpressed ENAH in several cancer types, and ENAH correlates with tumor invasiveness. This study aimed to investigate the expression and function of ENAH in primary gastric adenocarcinoma, and its prognostic significance. We found significantly increased mRNA (*P* = 0.0283) and protein (*P* = 0.0301) expression of ENAH in gastric cancer tissues. ENAH expression markedly associated with tumor size (*P* < 0.001), T stage (*P* < 0.001), N stage (*P* = 0.001), TNM stage (*P* < 0.001) and prognosis (*P* < 0.001). Cox regression analyses revealed ENAH expression as an independent predictor of overall survival (*P* = 0.019). We also analyzed data of 155 gastric cancer cases from The Cancer Genome Atlas (TCGA) and found that ENAH expression significantly correlated with age (*P* = 0.003), T stage (*P* = 0.023) and prognosis (*P* = 0.05). Furthermore, the function of ENAH in cell proliferation, colony formation, cell migration and invasion of gastric cancer cells was analyzed *in vitro*. Knockdown of ENAH expression suppressed cell proliferation, colony formation, cell migration and invasion in MKN45 cells. Conversely, overexpression of ENAH promoted cell proliferation, cell migration and invasion in MGC803 cells. Our research suggests that ENAH might play promoting functions in carcinogenesis and progression of gastric cancer, and may serve as a valuable prognostic marker for primary gastric adenocarcinoma patients.

## INTRODUCTION

Gastric cancer (GC) is one of the most common malignances and the leading cause of cancer-related death worldwide [1], with almost 1 million new cases and over 720,000 deaths reported in 2012 [2]. Statistics show that the overall clinical outcome for patients with advanced GC is poor, with 5-year survival rates of only about 5–20% and a median overall survival of 10 months [2, 3]. Despite developments in early detection and treatment (including radical surgery, chemotherapy, and radiotherapy), distant metastasis and local recurrence still occurs in most GC cases, and the clinical outcomes remains far from satisfactory [4–6].

GC results from a combination of environmental factors and the accumulation of generalized and specific genetic alterations, involving the activation of oncogenes and the inhibition of tumor suppressor genes [7]. Therefore, investigating the genetic alterations and molecular mechanisms involved in gastric carcinogenesis and progression will be critical for improving the diagnosis and treatment of GC [8].

ENAH, a member of the enabled (Ena)/vasodilator-stimulated phosphoprotein (VASP) family, is an actin regulatory protein involved in cell motility and adhesion [9, 10]. ENAH was recently reported to be up-regulated in many human cancers, including breast cancer and melanoma [11–13]. ENAH expression correlates with tumor grade and vascular invasion in salivary tumor [10]. In addition, ENAH expression is increased in hepatocellular carcinoma and is associated with tumor differentiation and clinical stage [14]. But thus far, the expression, clinical significance, and biological functions of ENAH in GC have not been explored.

In the present study, we analyzed ENAH expression levels in GC samples, and evaluated the functional role of ENAH in the tumorigenesis and progression of primary GC. We further identified the relationship between ENAH expression and clinicopathological features using a large GC population and evaluated its prognostic value in GC.

## RESULTS

### ENAH is overexpressed in human GC

**Figure 1:**
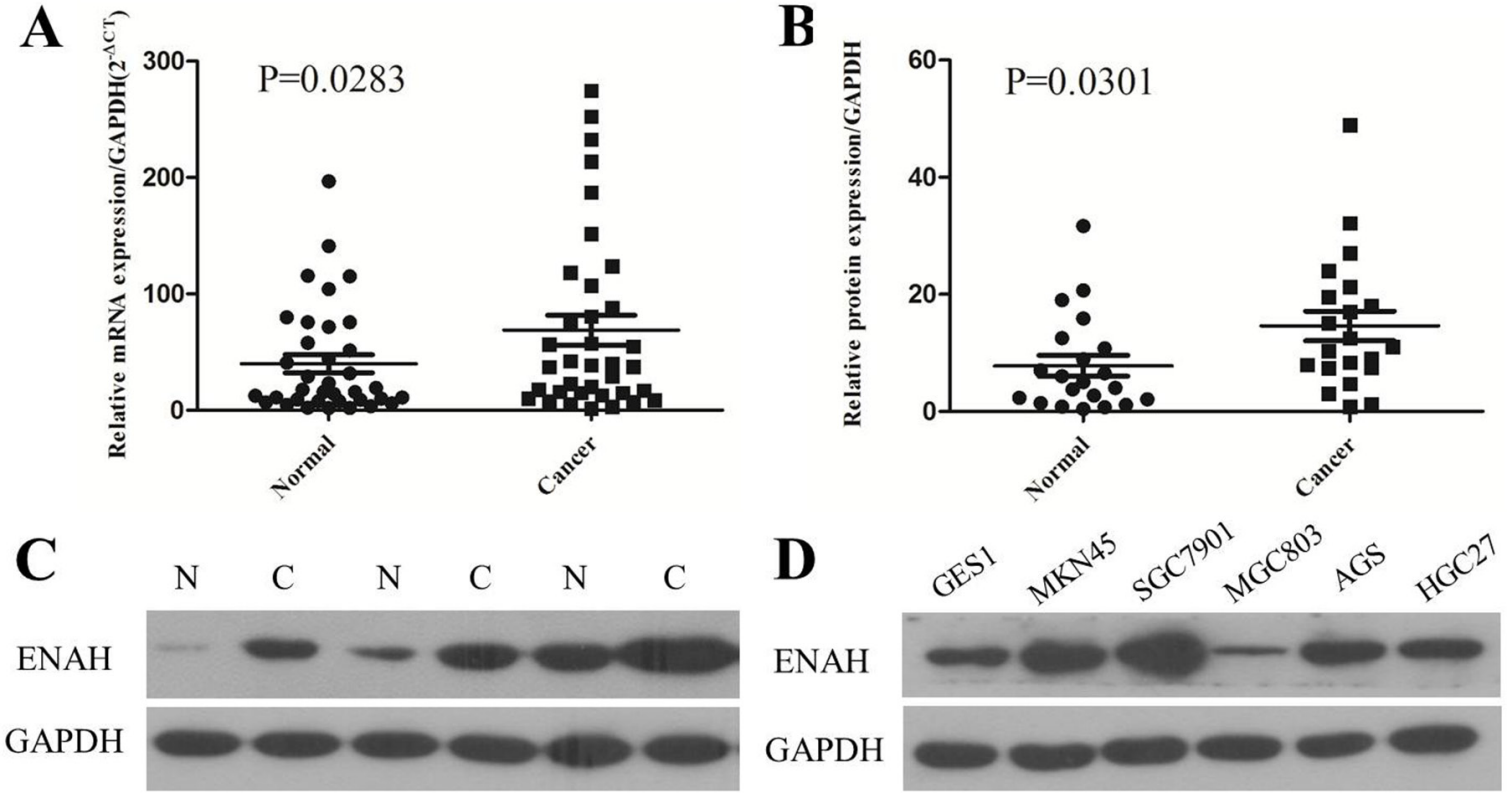
The mRNA and protein expression of ENAH is higher in human primary gastric adenocarcinoma surgical specimens and GC cell lines compared to controls **(A)** The relative mRNA expression of ENAH was significantly higher in GC tissues compared with the matched adjacent noncancerous tissues (n = 36, *P* = 0.0283). Horizontal lines represent the mean. **(B)** Relative ENAH protein expression was significantly higher in GC tissues compared with the matched adjacent noncancerous tissues (ENAH/GAPDH, n = 21, *P* = 0.0301). Horizontal lines represent the mean. **(C)** Representative results of ENAH protein expression in three paired GC tissues and matched adjacent noncancerous tissues (N, matched noncancerous gastric mucosa; C, GC tissues). **(D)** ENAH protein expression in the normal gastric cell line GES1 and in GC cell lines MKN45, SGC7901, MGC803, AGS, and HGC27.

To evaluate the expression pattern of ENAH in GC, we examined mRNA levels by real-time quantitative PCR (qRT-PCR) in 36 human primary GC and matched adjacent normal mucosa tissues. The ENAH mRNA expression level was significantly higher in 22 (61.1%) GC tissues compared with the adjacent non-tumor tissues (*P* = 0.0283, Figure 1A).

Consistent with the results of qRT-PCR, our western blot analyses revealed higher protein expression of ENAH in 76.2% (16/21) of GC tissues than their matched non-cancerous tissues (*P* = 0.0301, Figure 1B and 1C). Likewise, the ENAH protein expression was remarkably higher in MKN45, SGC7901, AGS, and HGC27 cell lines compared with the normal gastric cell line GES1 (Figure 1D).

### The role of ENAH in proliferation and colony formation in GC cells

**Figure 2:**
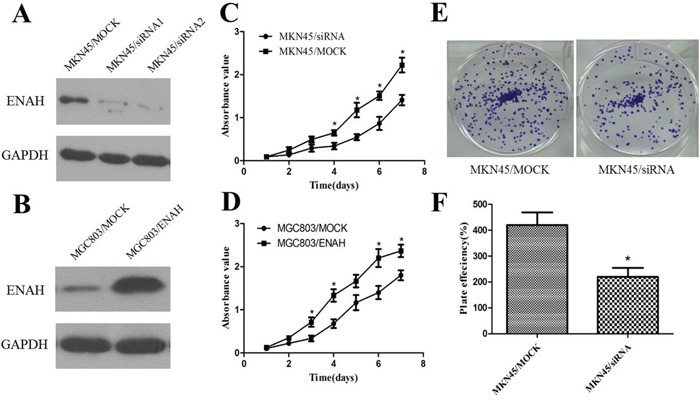
ENAH promotes proliferation and colony formation in MKN45 and MGC803 cell lines **(A)** Western blot analysis of silenced ENAH expression in MKN45 cells. **(B)** Western blot analysis of ENAH overexpression in MGC803 cells. **(C)** Cell-proliferation assay showing that silencing ENAH expression inhibited proliferation of MKN45 cells. **(D)** Cell-proliferation assay showing that ENAH overexpression promoted proliferation of MGC803 cells. **(E)** Representative results showing silencing ENAH expression inhibited colony formation of MKN45 cells. **(F)** Quantitative analyses of foci numbers in Figure 2E shown as the mean ± SD. * *P* < 0.05 versus control.

To evaluate the effects of ENAH on cell proliferation, we silenced ENAH expression in the MKN45 cell line with siRNA and then detected the level of ENAH expression in transfected cells by western blotting (Figure 2A). Silencing the expression of ENAH in MKN45 cells significantly inhibited cell proliferation compared with mock siRNA treatment (Figure 2C). Meanwhile, the efficiency of colony formation was significantly (*P* = 0.0294) suppressed in ENAH-specific siRNA transfected GC cells compared with mock siRNA transfected GC cells (Figure 2E and 2F).

ENAH protein expression was lower in MGC803 cells compared with GES1 cells (Figure 1D). Therefore, to further confirm the function of ENAH, we constructed an ENAH expression vector (pDC316-mCMV-ENAH) and transfected MGC803 cells (Figure 2B). The cell growth rate was significantly enhanced in MGC803 cells overexpressing ENAH (Figure 2D).

### ENAH promotes cell migration and invasion in GC cells

**Figure 3:**
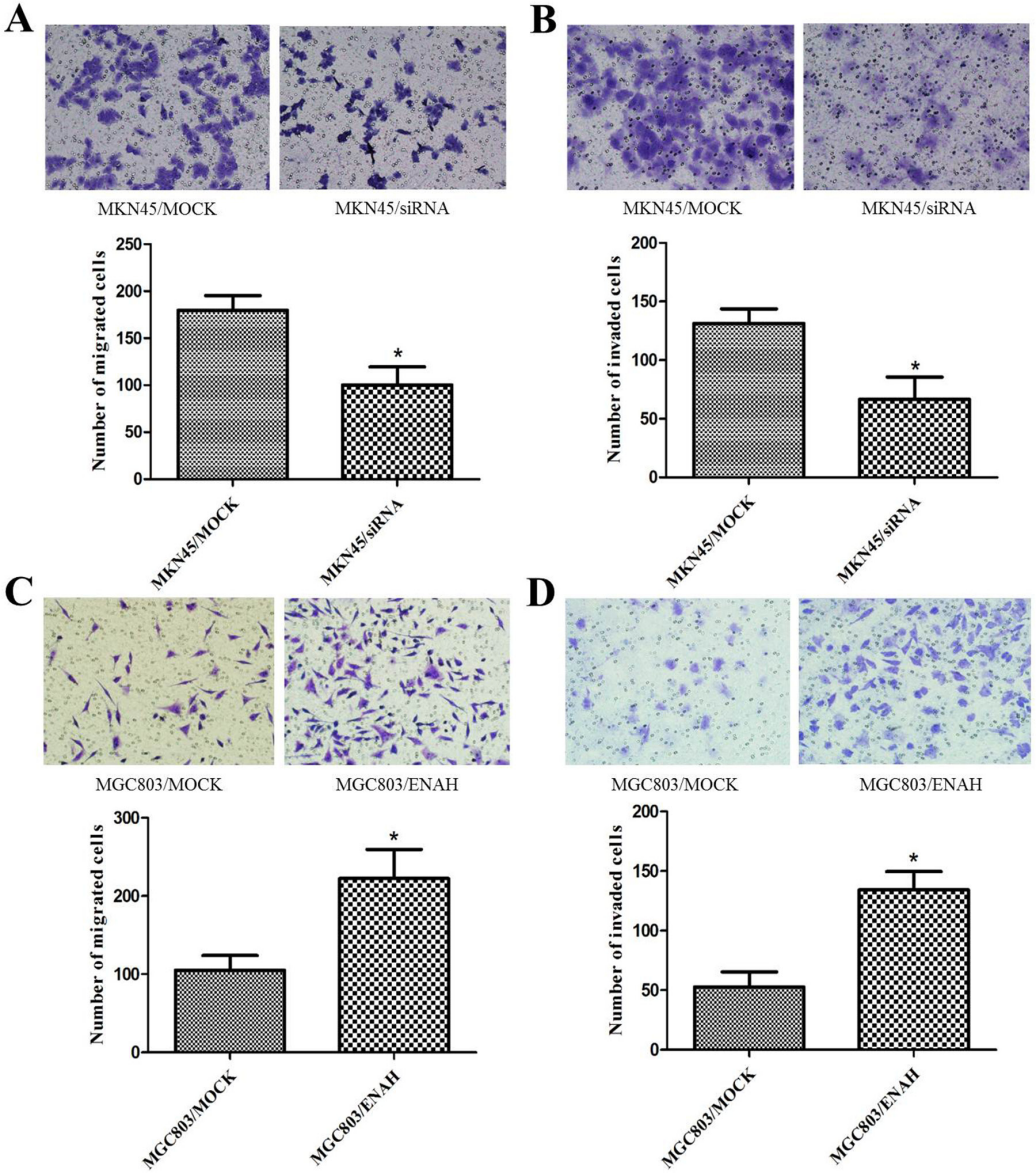
ENAH promotes cell migration and invasion in MKN45 and MGC803 cells Images (upper panel) of the transwell migration and matrigel invasion assays are shown using ×100 magnification; ten randomly selected fields were used for quantification (lower panel, bar graphs). Values are expressed as the mean ± SD of three independent experiments. **(A, B)** ENAH knockdown inhibited migration (A) and invasion (B) of MKN45 cells. **(C, D)** ENAH overexpression promoted migration (C) and invasion (D) of MGC803 cells. * *P* < 0.05 versus control.

We conducted transwell assay to evaluate the effects of ENAH expression on GC cell migration and invasion. Silencing ENAH expression in MKN45 cells resulted in a significant decrease in the number of cells that migrated or invaded through the membrane in the transwell chamber (Figure 3A and 3B). Conversely, overexpression of ENAH significantly enhanced the migration and invasion of MGC803 cells compared with MGC803 cells transfected with a control vector (Figure 3C and 3D). These results suggest that increased ENAH expression may further promote GC progression.

### Immunohistochemical analysis of ENAH expression in GC tissue samples and its relationship with clinicopathological parameters

**Figure 4:**
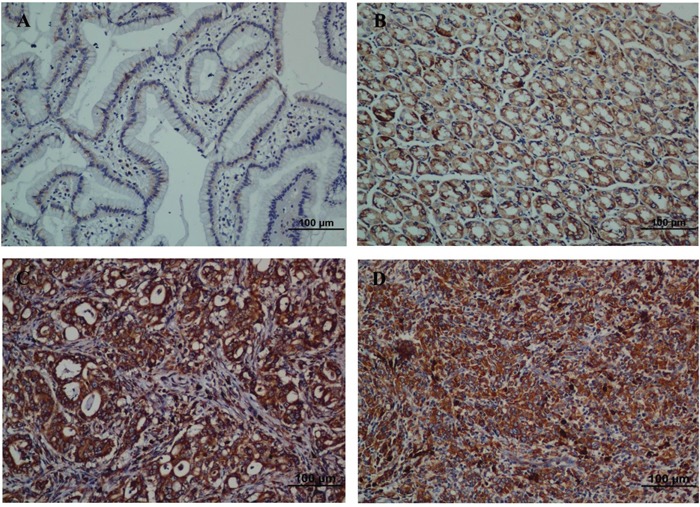
Strong expression of ENAH protein is observed in GC surgical specimens by immunohistochemistry **(A)** Weak ENAH staining was observed in noncancerous gastric mucosa. **(B)** Weak ENAH staining was observed in well-differentiated GC. **(C)** Strong ENAH staining was observed in moderately differentiated GC. **(D)** Strong ENAH staining was observed in poorly differentiated GC.

To further elucidate the clinicopathological and prognostic roles of ENAH expression, we carried out immunohistochemical analyses of the 238 paraffin-embedded GC tissues. Among the 238 GC tissue samples, we found 102 (42.9%) cases with low ENAH expression and 136 (57.1%) cases with high ENAH expression. Noncancerous gastric mucosa and well-differentiated gastric adenocarcinoma showed low ENAH expression (Figure 4A and 4B). Moderately and poorly differentiated gastric adenocarcinoma showed high ENAH expression (Figure 4C and 4D).

Chi-square analyses revealed that ENAH expression was significantly correlated with tumor size (*P* < 0.001), depth of tumor infiltration (T stage, *P* < 0.001), local lymph node metastasis (N stage, *P* = 0.001), and TNM stage (*P* < 0.001), but not with age, gender, and distant metastasis (M stage). The correlations between ENAH expression and clinicopathological parameters are listed in Table 1.

**Table 1:** Correlation between ENAH expression and clinicopathological variables of 238 gastric cancer cases

Clinicopathological parameters	*n*^a^	ENAH expression		χ^2^	*P* value
		High	Low		
**All**	238	136	102		
**Age (years)**					
<55	116	65	51	0.114	0.736
≥55	122	71	51		
**Gender**				1.021	0.312
Male	87	46	41		
Female	151	90	61		
**Tumor size**				15.025	<0.001*
<3 cm	51	17	34		
≥3 cm	187	119	68		
**Tumor infiltration**				39.028	<0.001*
T1	41	10	31		
T2	37	13	24		
T3	4	2	2		
T4a	111	82	29		
T4b	45	29	16		
**Local lymph node metastasis**				16.250	0.001*
N0	84	36	48		
N1	44	23	21		
N2	37	23	14		
N3	73	54	19		
**Distant metastasis**				0.015	0.903
M0	213	122	91		
M1	25	14	11		
**TNM staging**				40.926	<0.001*
1	48	9	39		
2	96	61	35		
3	65	50	15		
4	29	16	13		

^a^ Numbers of cases in each group. * Statistically significant (*P*<0.05).

### ENAH expression and patient survival

**Figure 5:**
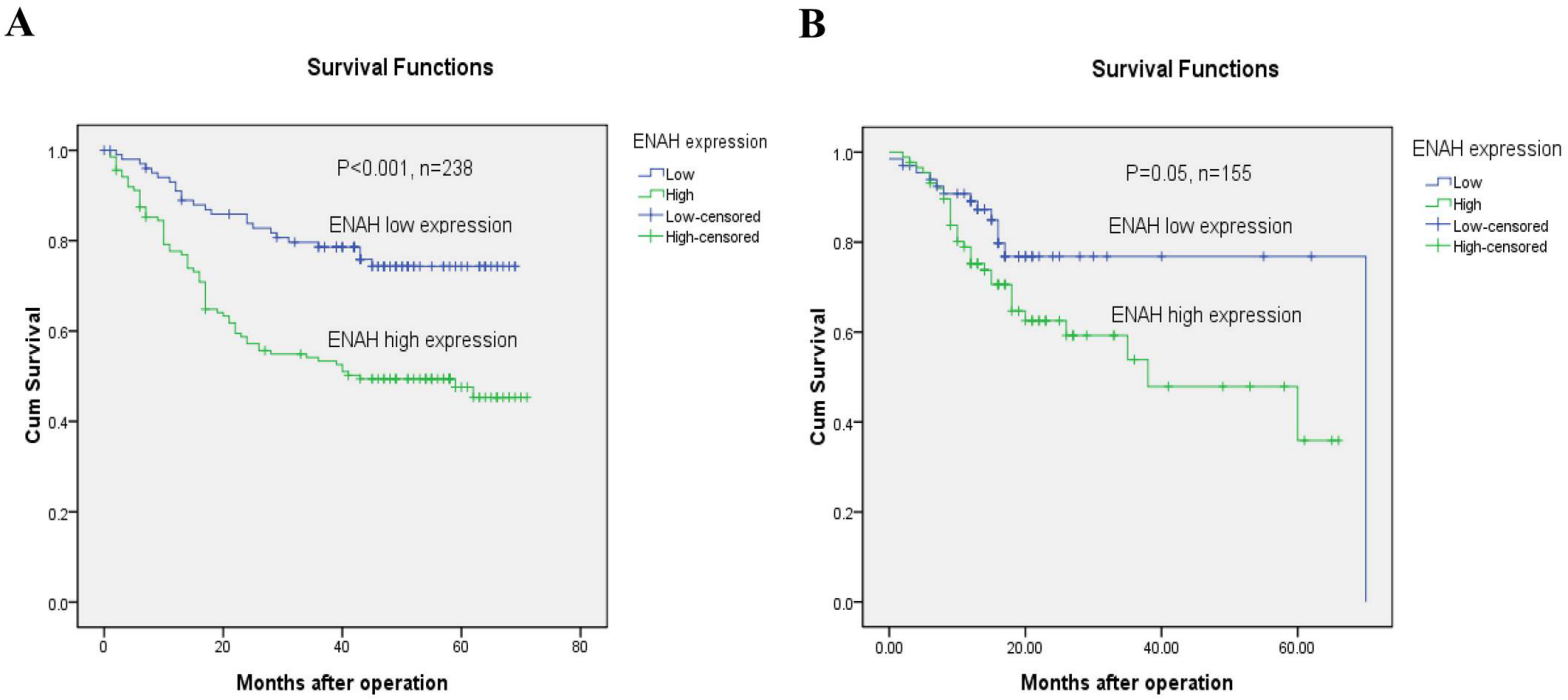
GC patients with high ENAH show lower survival than those with low expression **(A)** Kaplan-Meier survival curves of GC patients (n = 238) after gastrectomy. The survival rate of patients in high ENAH expression group was significantly lower than that of patients in low ENAH expression group (log-rank test, *P* < 0.001). **(B)** Kaplan-Meier survival curves for the TCGA gastric cancer cases (n = 155). The survival rate of patients in high ENAH expression group was significantly lower than that of patients in low ENAH expression group (log-rank test, *P* = 0.05).

The 5-year overall survival rates in patients with low and high ENAH expression were 71.5% and 47.6%, respectively. Kaplan-Meier analyses revealed that the overall survival of GC patients with high ENAH expression was significantly worse than that of patients with low ENAH expression (*P* < 0.001, log-rank test, Figure 5A).

### Univariate and multivariate analyses of prognostic variables in GC patients

Further univariate and multivariate analyses were performed using a Cox proportional-hazards model to investigate the effect of ENAH expression and other clinicopathological parameters in GC patients. Univariate Cox regression analyses showed that tumor size (*P* < 0.001), T stage (*P* < 0.001), N stage (*P* < 0.001), M stage (*P* < 0.001), TNM stage (*P* < 0.001) and ENAH expression (*P* < 0.001) were significant prognostic factors (Table 2). A multivariate Cox regression analysis confirmed tumor size (*P* = 0.036), TNM stage (*P* < 0.001), and ENAH expression (*P* = 0.019) as independent prognostic predictors for GC patients (Table 2).

**Table 2:** Univariate and multivariate analyses of overall survival of gastric cancer patients

Variables	*n*^a^	Univariate analyses			Multivariate analyses		
		HR	(95% CI)	*P* value	HR	(95% CI)	*P* value
**Age (years)**				0.217			0.187
<55	116	1.000			1.000		
≥55	122	1.295	0.859-1.953		1.330	0.870-2.033	
**Gender**				0.468			
Male	87	1.000			1.000		0.923
Female	151	1.170	0.765-1.790		1.022	0.660-1.584	
**Tumor size**				<0.001*			0.036*
<3 cm	51	1.000			1.000		
≥3 cm	187	9.835	3.112-31.083		3.633	1.088-12.126	
**Tumor infiltration**				<0.001*			
T1-2	78	1.000					
T3-4	160	19.562	6.188-61.837				
**Local lymph node metastasis**				<0.001*			
N0	84	1.000					
N1	44	4.452	1.815-10.921				
N2	37	6.940	2.898-16.619				
N3	73	13.052	5.923-28.760				
**Distant metastasis**				<0.001*			
M0	213	1.000					
M1	25	5.397	3.301-8.823				
**TNM staging**				<0.001*			<0.001*
1-2	144	1.000			1.000		
3-4	94	6.222	3.898-9.931		4.339	2.655-7.090	
**ENAH**				<0.001*			0.019*
Low	102	1.000			1.000		
High	136	2.566	1.612-4.086		1.764	1.098-2.835	

HR, hazard ratio; CI, confidence interval; ^a^ Numbers of cases in each group; * Statistically significant (*P* < 0.05).

### Clinicopathological and prognostic analyses with TCGA clinical GC data

We analyzed data of 155 TCGA GC cases downloaded from the UCSC Cancer Genomics Browser database. Among the 155 TCGA GC cases, we found 67 (43.2%) cases with low ENAH expression and 88 (56.8%) cases with high ENAH expression (Table 3). ENAH expression was significantly correlated with depth of tumor infiltration (T stage, *P* = 0.023, Table 3) and age (*P* = 0.003, Table 3).

**Table 3:** Correlation between ENAH expression and clinicopathological variables of 155 TCGA gastric cancer cases

Clinicopathological parameters	*n*^a^	ENAH expression		χ^2^	*P* value
		High	Low		
**All**	155	88	67		
**Age (years)**					
<55	31	25	6	8.997	0.003*
≥55	124	63	61		
**Gender**				0.015	0.903
Male	98	56	42		
Female	57	32	25		
**Tumor infiltration**				5.183	0.023*
T1-2	35	14	21		
T3-4	120	74	46		
**Local lymph node metastasis**				0.044	0.834
N0-1	94	54	40		
N2-3	61	34	27		
**Distant metastasis**				0.015	0.903
M0	148	84	64		
M1	7	4	3		
**TNM staging**				1.894	0.595
1	22	10	12		
2	54	30	24		
3	68	42	26		
4	11	6	5		

^a^ Numbers of cases in each group. * Statistically significant (*P*<0.05).

Kaplan-Meier analyses revealed that the overall survival of GC patients with high ENAH expression was significantly worse than that of patients with low ENAH expression (*P* = 0.05, log-rank test, Figure 5B). Univariate Cox regression analyses showed that N stage (*P* = 0.049), M stage (*P* = 0.002), and TNM stage (*P* = 0.042) were significant prognostic factors, and the *P* value for ENAH expression was 0.056 (Table 4). TNM stage (*P* = 0.037) was confirmed as a significant independent predictor by multivariate Cox regression analysis (Table 4).

**Table 4:** Univariate and multivariate analyses of overall survival of 155 TCGA gastric cancer patients

Variables	*n*^a^	Univariate analyses			Multivariate analyses		
		HR	(95% CI)	*P* value	HR	(95% CI)	*P* value
**Age (years)**				0.330			0.344
<55	31	1.000			1.000		
≥55	124	1.499	0.664-3.385		1.484	0.656-3.357	
**Gender**				0.956			
Male	98	1.000			1.000		0.640
Female	57	0.983	0.532-1.817		0.861	0.459-1.614	
**Tumor infiltration**				0.064			
T1-2	35	1.000					
T3-4	120	2.279	0.953-5.451				
**Local lymph node metastasis**				0.049*			
N0-1	94	1.000					
N2-3	61	1.818	1.004-3.292				
**Distant metastasis**				0.002*			
M0	148	1.000					
M1	7	5.213	1.820-14.932				
**TNM staging**				0.042*			0.037*
1	22	1.000			1.000		
2	54	1.555	0.503-4.804		1.433	0.462-4.444	
3	68	2.064	0.703-6.057		1.981	0.675-5.809	
4	11	5.245	1.474-18.667		5.313	1.474-19.156	
**ENAH**				0.056			
Low	67	1.000					
High	88	1.914	0.984-3.724				

HR, hazard ratio; CI, confidence interval; ^a^ Numbers of cases in each group; * Statistically significant (*P* < 0.05).

## DISCUSSION

The Ena/VASP family comprises three members: Ena-VASP-like (Evl), Mena (ENAH), and VASP. These proteins have similar tripartite domain organization consisting of an N-terminal Ena/VASP homology 1 (EVH1) domain and a variable central proline-rich region, followed by a C-terminal EVH2 domain [15, 16]. The EVH1 domain is responsible for targeting Ena/VASP proteins to the leading edge of cells to control cell migration and motility, and the EVH2 domain regulates actin polymerization [17].

While ENAH expression was recently reported to be increased in breast cancer, hepatocellular carcinoma, and melanoma [10–13], its role in GC remained undefined. In the current study, we found mRNA and protein expression of ENAH were significantly higher in primary GC tissues compared with adjacent non-tumor tissues. Likewise, the ENAH protein expression was remarkably higher in MKN45, SGC7901, AGS and HGC27 cell lines, compared with normal gastric cell line GES1. ENAH was also found to be highly expressed in 136 out of 238 (57.1%) GC samples, with lower expression in another 102 (42.9%) cases. Finally, analysis of 155 samples from the TCGA databases revealed 67 of 155 (43.2%) GC samples with low ENAH expression and 88 (56.8%) cases with high ENAH expression.

We then explored the role of ENAH in the proliferation and colony formation of GC *in vitro*. We found that silencing ENAH expression in MKN45 cells significantly inhibited cell proliferation and colony formation, whereas ENAH overexpression in MGC803 cells significantly enhanced cell growth rate and colony formation. These results indicated that ENAH may promoting tumor cell growth.

ENAH protein has been shown to have several splice variants, including the hMena^INV^ and hMena^11a^ isoforms [18–20]. The hMena^INV^ isoform is reportedly expressed exclusively in invasive cancer cells [19, 20], while the hMena^11a^ isoform is expressed specifically in epithelia in primary breast carcinomas and is downregulated in invasive tumor cells [19, 20]. Tanaka et al. found that hMena^INV^ expression is augmented during tumor progression in breast cancer, and the relative expression of hMena^11a^ compared with hMena^INV^ is linked to malignant transformation in breast epithelial cells and cancer progression [10]. Therefore, in the present study, we explored the role of ENAH in cell migration and invasion in GC cells. Silencing ENAH expression significantly inhibited the migration and invasion in MKN45 cells, while ENAH overexpression significantly enhanced the migration and invasion in MGC803 cells. Furthermore, immunohistochemical analyses of clinical GC paraffin specimens showed that ENAH expression was significantly associated with tumor infiltration and local lymph node metastasis. These results suggest that increased expression of ENAH may further promote GC progression.

ENAH expression was previously reported to correlate with tumor grade and vascular invasion in salivary tumors [9], and is associated with tumor differentiation and clinical stage hepatocellular carcinoma [13]. Herein, in a large GC population (238 cases), we found that the expression of ENAH was significantly correlated with tumor size (*P* < 0.001), tumor infiltration (*P* < 0.001), local lymph node metastasis (*P* = 0.001), and TNM stage *(P* < 0.001). In addition, we detected higher ENAH immune reactivity in poorly differentiated GC tissues than in well-differentiated ones, suggesting that amplified ENAH expression might correlate with GC dedifferentiation. TCGA data analyses also showed that ENAH expression was significantly correlated with the depth of tumor infiltration (*P* = 0.023), further supporting our proposal that ENAH plays important roles in the progression and dedifferentiation of GC.

Importantly, Kaplan-Meier survival analyses revealed that higher expression of ENAH was significantly correlated to worse overall survival than lower ENAH expression. TCGA data analyses also support our results. Cox regression analyses further demonstrated ENAH expression as an independent prognostic factor for GC patients. This finding indicates that high ENAH expression might be useful for classifying GC patients with a poor prognosis and provides further evidence that ENAH may promote the progression of GC. However, it is important to note that the follow-up time used in our study was relatively short, and as such, no deaths were observed among early stage (TNM stage I) GC patients. We will continue to follow-up this cohort of patients and perform further statistical analyses on survival rates in future.

With regards to its molecular mechanism of action in tumorigenesis and progression, previous studies have shown that ENAH promotes actin polymerization at the leading edge of migrating cells [15–17]. In addition, Trono et al. found the hMENA^11a^ isoform sustains cell proliferation and survival in HER2-overexpressing breast cancer cells primarily through activating the HER3/AKT axis, and contributes to HER3-mediated resistance mechanisms to PI3K inhibitors [21]. However, the molecular mechanisms of ENAH in GC require thorough investigation in future.

In conclusion, we have demonstrated amplified expression of ENAH in gastric adenocarcinoma and its correlation with a more malignant phenotype and unfavorable prognosis in a large number of clinical GC samples. We confirmed that ENAH enhance GC cell growth, colony formation, cell migration, and invasion *in vitro*. Taken together, our research suggests that ENAH might serve as a candidate prognostic biomarker for GC patients and a potential target for gene therapy in the treatment of GC patients.

## MATERIALS AND METHODS

### Ethics statement

The research was approved by the Ethics Committee of Shandong Academy of Medical Sciences and Ethics Committee of Sun Yat-sen University Cancer Center. Written informed consent was obtained from each patient involved in the study.

### Cell lines and culture

The GC cell lines MKN45, SGC7901, and MGC803 were obtained from the Committee of Type Culture Collection of Chinese Academy of Sciences (Shanghai, China). All cells were cultured in RPMI 1640 medium containing 10% heat-inactive fetal bovine serum (FBS, Gibco, Grand Island, NY). The cells were incubated at 37°C in a humidified 5% CO_2_ atmosphere.

### Tissue samples

GC tissues and adjacent noncancerous gastric tissue samples were collected from 36 primary GC patients undergoing radical gastrectomy at Sun Yat-sen University Cancer Center between 2010 and 2012. None of the patients had been treated before surgery. Fresh tissues were immediately immersed in RNA later (Ambion, Inc., USA) to avoid RNA degradation, and then stored at 4°C overnight. All samples were subsequently frozen at −80°C until RNA and protein extraction was performed.

### GC patients and follow-up

Paraffin-embedded primary GC samples were obtained from 238 postoperative patients in Sun Yat-sen University Cancer Center between January 2003 and December 2006. All patients in our study belonged to the same ethnic group. The patients were selected according to the criteria: (1) histopathological identification of gastric adenocarcinoma; (2) limited or extended surgical history including gastrectomy plus lymphadenectomy; (3) no chemotherapy and radiotherapy before surgery; (4) complete follow-up data; (5) no history of other synchronous malignancies or familial malignancy; (6) no recurrent GC or remnant GC; and (7) no death in the perioperative period. The surgery was performed by experienced surgeons following the Japanese Gastric Cancer Association (JGCA) guidelines.

The TCGA clinical GC data were downloaded from the UCSC Cancer Genomics Browser database (https://genome-cancer.ucsc.edu/). The 155 GC patients with overall follow-up data were selected according to the criteria described above.

Postoperative follow-up of 238 patients from Sun Yat-sen University Cancer Center was conducted every 3 months for the first 2 years, every 6 months during the third to fifth years, and then annually for an additional 5 years or until patient death, whichever occurred first. The characteristics of these patients are listed in Table 1. The Tumor-Node-Metastasis (TNM) stage was recorded based on the 7^th^ edition of the International Union Against Cancer (UICC).

### Real-time qRT-PCR

Total RNA was extracted from fresh tissue samples by TRIzol Reagent (Invitrogen, Carlsbad, CA) according to manufacturer's instructions. The concentration and quality of total RNA was assessed using a Nanodrop Spectrophotometer (ND-1000; Thermo Scientific) by measuring the absorbance at 260 nm. The cDNA was synthesized through reverse transcription (RT) with 2 μg total RNA in a 25 μL reaction system using M-MLV Reverse Transcriptase (Promega, Fitchburg, WI). The reaction system was incubated at 70°C for 5 min, 42°C for 1 h. The resulting cDNA was subjected to real-time qRT-PCR analyses to evaluate the relative mRNA levels of ENAH and GAPDH (glyceraldehyde-3-phosphate dehydrogenase, as an internal control) in primary GC tumors compared to the paired noncancerous gastric tissues.

Gene-specific amplification was carried out using an ABI 7900HT Real-time PCR system (Life Technologies, Carlsbad, CA) with a 15 μL reaction system containing 0.5 μl cDNA, 7.5 μl 2× SYBR Green master mix (Invitrogen, Carlsbad, California, USA), and 200 nM of the appropriate oligonucleotide primers. All measurements were performed in triplicate, after undergoing the following reaction cycle: preheat at 95°C for 10 min, 40 cycles of 95°C for 30 sec, and 60°C for 1 min. The melting curve was measured at 95°C for 15 sec, 60°C for 15 sec, and 72°C for 15 sec. The Ct (threshold cycle) value of each sample was measured during exponential amplification, and was calculated from threshold cycles with the software SDS 2.3. Data were analyzed using the comparative threshold cycle method (2^−ΔCT^). Relative expression levels of ENAH were normalized to the geometric mean of the internal control GAPDH.

Primers for real-time PCR were: ENAH forward 5′-TCAAGGGTAAGGGAAACTGG-3′, and reverse 5′-TGGCTCACAAGTGGTCCTCC-3′; GAPDH forward 5′-CTCCTCCTGTTCGACAGTCAGC-3′, and reverse 5′-CCCAATACGACCAAATCCGTT-3′.

### Western blot analysis

Total protein of cell or tissue lysate was isolated as previously described [22]. About 30 μg of the protein extraction was separated by sodium dodecyl sulfate polyacrylamide gel electrophoresis and then transferred to a polyvinylidene fluoride membrane. After blocking the nonspecific binding sites for 60 min with 5% non-fat milk, the membrane was incubated with a rabbit monoclonal antibody against ENAH (Cell Signaling Technology, at 1000 dilution) at 4°C overnight. Membranes were washed three times with TBST (tris buffered saline with 1‰ tween-20) and incubated with horseradish peroxidase (HRP)-conjugated goat anti-rabbit antibody (1:5000 dilution; AQ132P, Merck, White house Station, NJ) at 37°C for 1 h.

After 3 washes, the bands were detected by an enhanced chemiluminescence system (Cell Signaling Technology, Danvers, Massachusetts, USA). The internal control GAPDH was detected using a HRP-conjugated mouse anti-human GAPDH monoclonal antibody (Shanghai Kangchen, China, at 1:5000 dilution). Band density was measured with ImageJ software (National Institutes of Health, Bethesda, MD) and was standardized to that of GAPDH.

### Expression plasmids and transient transfections

A eukaryotic expression plasmid pDC316-mCMV-EGFP, containing the full-length human Mena cDNA, was obtained from the Land Biology Company (Guangzhou, China). Empty vector was used as a negative control. GC cells were cultured in 6-well plates until they reached 85–90% confluence. Transient transfections were performed using Lipofectamine 2000 (Invitrogen), according to the manufacturer's instructions. At 48 h after transfection, protein expression was examined by western blotting.

### RNA oligonucleotides and cell transfections

Small interfering RNA (siRNA, 20 μM, synthesized by GenePharma Company, Shanghai, China) was transfected into GC cells using Lipofectamine RNAi MAX (Invitrogen) according to the manufacturer's instructions. The siRNA sequences were: siRNA-ENAH#1, sense 5′-GGUCCUAUGAUUCAUUACATT-3′, and antisense 5′-UGUAAUGAAUCAUAGGACCTT-3′; siRNA-ENAH#2, sense 5′-GCGAGAAAGAAUGGAAAGATT-3′, and antisense 5′- UCUUUCCAUUCUUUCUCGCTT-3′; negative control (NC), sense 5′-UUCUCCGAACGUGU CACGUTT-3′, and antisense 5′-ACGUGACACGUUCG GAGAATT-3′. After transfection for 48 h, protein expression was examined by western blotting.

### Proliferation assay

The proliferation rate of cells was evaluated using the MTS Cell Proliferation kit (Promega, Beijing, China) according to manufacturer's instructions. Cells were seeded in 96-well plates (5×10^2^ per well), and each experiment was done in triplicate.

### Colony formation assay

Cells were seeded in six-well plates (500 cells/well) and cultured at 37°C in an atmosphere of 5% CO_2_ for 10 days. Surviving colonies (>50 cells per colony) were washed twice with PBS, fixed in 75% alcohol for 30 min, and stained with 0.5% (m/v) crystal violet for 30 min. Colony-forming efficiency (CFE%) was defined as the ratio of the number of colonies formed in culture to the number of cells inoculated. Three independent experiments were performed.

### Migration and invasion assays

Migration and invasion assays were performed in Transwell chambers (8-μm pores, Corning, Shanghai, China) placed in 24-well plates. For invasion assay, the bottom of the Transwell chamber was coated with a thin layer of 0.5 mg/ml Matrigel Basement Membrane Matrix (BD Biosciences, Bedford, MA, USA). Cells in 150 μL RPMI 1640 (2×10^5^ cells/mL for migration assay or 1×10^6^ cells/mL for invasion assay) without FBS were added into the upper Transwell chamber, and 500 μL RPMI 1640 containing 20% FBS was placed in the lower chamber. The cells were incubated at 37°C and allowed to migrate or invade through the Matrigel layer. After 48 h, the cells were fixed with 75% methanol for 10 min. The migrated or invaded cells on the lower surface of Transwell chamber were stained with 0.5% (m/v) crystal violet for 1 h. The stained cells were counted in 10 random fields under an inverted microscope. Each experiment was carried out in three separate wells, and independent experiments were repeated at least three times.

### Immunohistochemistry and semi-quantitative analysis

Immunohistochemistry analysis was performed as previously described [22]. The antibodies were: a rabbit monoclonal antibody against ENAH (Cell Signaling Technology, at 500 dilution), and a biotinylated secondary antibody (Zhongshan Golden Bridge Biotech, Beijing, China).

The ENAH protein expression level was assessed by an immunostaining score, calculated as the sum of the percent positivity of stained tumor cells and the staining intensity, and ranged from 0 to 6. Staining intensity was scored as: 0 (no staining), 1 (weak staining, visible at high magnification), 2 (moderate staining, visible at low magnification), and 3 (dark staining, strikingly positive at low magnification). The percentage of positive staining was scored as: 0 (0–9%, negative), 1 (10%–25%, sporadic), 2 (26%–50%, focal), or 3 (51%–100%, diffuse). ENAH expression was defined as: “-” (negative; score 0), “+” (weakly positive; score 1–2), “++” (positive; score 3–4), “+++” (strongly positive; score 5–6). Based on the ENAH expression levels, we divided the GC patients into two groups: low ENAH expression (ENAH “-” or ENAH “+”) and high ENAH expression (ENAH “++” or ENAH “+++”).

The 155 TCGA patients were divided into two groups: low ENAH expression (ENAH expression value < 3.0000) and high ENAH expression (ENAH expression value ≥ 3.0000).

### Statistical analysis

Statistical analyses were carried out with the Statistical Package for the Social Sciences, version 17.0 (SPSS Inc., Chicago, IL, USA). Differences between levels of mRNA and protein expression in tumor samples and their paired non-tumor tissue samples were assessed using the paired-sample *t* test. ANOVA analysis was used to detect significant differences in the cell proliferation curves. The relationships between ENAH expression and various clinicopathological parameters were assessed using the chi-squared test. Survival curves were plotted using the Kaplan-Meier method and groups compared using the log-rank test. The Cox proportional hazard regression model was used for univariate and multivariate analyses to identify the effects of clinicopathological variables of GC and ENAH expression on survival. The results were expressed as mean ± standard deviation (SD), and a two-sided *P* value less than 0.05 was considered to be statistically significant.
